# Differential stepwise evolution of SARS coronavirus functional proteins in different host species

**DOI:** 10.1186/1471-2148-9-52

**Published:** 2009-03-05

**Authors:** Xianchun Tang, Gang Li, Nikos Vasilakis, Yuan Zhang, Zhengli Shi, Yang Zhong, Lin-Fa Wang, Shuyi Zhang

**Affiliations:** 1 School of Life Science, East China Normal University, Shanghai, 200062, PR China; 2 Current address: Center for Vaccine Research, University of Pittsburgh, Pennsylvania, 15261, USA; 3 School of Life Sciences, Fudan University, Shanghai, 200433, PR China; 4 Institute of Virology, Chinese Academy of Sciences, Wuhan, 430071, PR China; 5 CSIRO Livestock Industries, Australian Animal Health Laboratory and Australian Biosecurity Cooperative Research Centre, Geelong, Victoria 3220, Australia

## Abstract

**Background:**

SARS coronavirus (SARS-CoV) was identified as the etiological agent of SARS, and extensive investigations indicated that it originated from an animal source (probably bats) and was recently introduced into the human population via wildlife animals from wet markets in southern China. Previous studies revealed that the spike (S) protein of SARS had experienced adaptive evolution, but whether other functional proteins of SARS have undergone adaptive evolution is not known.

**Results:**

We employed several methods to investigate selective pressure among different SARS-CoV groups representing different epidemic periods and hosts. Our results suggest that most functional proteins of SARS-CoV have experienced a stepwise adaptive evolutionary pathway. Similar to previous studies, the spike protein underwent strong positive selection in the early and middle phases, and became stabilized in the late phase. In addition, the replicase experienced positive selection only in human patients, whereas assembly proteins experienced positive selection mainly in the middle and late phases. No positive selection was found in any proteins of bat SARS-like-CoV. Furthermore, specific amino acid sites that may be the targets of positive selection in each group are identified.

**Conclusion:**

This extensive evolutionary analysis revealed the stepwise evolution of different functional proteins of SARS-CoVs at different epidemic stages and different hosts. These results support the hypothesis that SARS-CoV originated from bats and that the spill over into civets and humans were more recent events.

## Background

Severe acute respiratory syndrome (SARS) emerged in Guangdong province of China in November 2002 and subsequently spread rapidly to 25 countries across five continents within 3–4 months [1]. Soon after its first outbreak, the etiological agent of SARS was identified as a novel coronavirus [2-4], and its complete genome sequence was determined [3,5,6]. The identification of SARS-CoV in Himalayan palm civets and raccoon dogs in live animal markets in Guangdong, China, provided the first clue of an animal-to-human transmission [7,8]. Further studies indicated that civets were unlikely to be the natural reservoir [9]. Instead the detection of different SARS-like-CoVs in horseshoe bats (*Rhinolophus spp*.) seemed to suggest that bats might be the natural reservoir of SARS-CoV and many other closely related coronaviruses [10-13].

**Figure 1:**
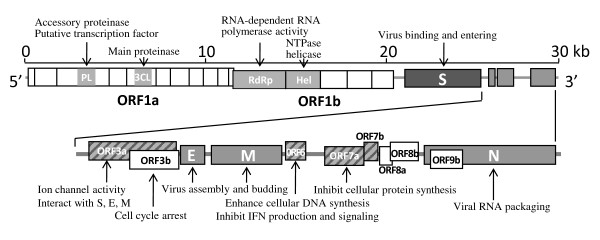
**Schematic diagram of the SARS-CoV genome organization and viral proteins**. The protein coding regions analyzed in this paper were shaded in grey.

Like other coronaviruses, SARS-CoV is an enveloped, positive-stranded RNA virus with a genome of approximately 29,700 nucleotides. The genome contains at least 14 open reading frames (ORFs) that encode 28 proteins in three distinct classes: two large polyproteins P1a and P1ab that are cleaved into 16 non-structural proteins (nsp1–nsp16) during viral RNA synthesis; four structural proteins (S, E, M and N) that are essential for viral entry and assembly; and eight accessory proteins that are believed to be non-essential for viral replication, but may facilitate viral assembly and play a role in viral virulence and pathogenesis (Figure 1) [14-17].

Similar to all RNA viruses, SARS-CoV replication is associated with genomic and antigenic variation. The ω ratio (*d*_N_/*d*_S _ratio of non-synonymous to synonymous substitutions) can measure the selective pressure at protein level, with ω = 1, < 1, > 1 indicating neutral selection, negative selection and positive selection, respectively [18,19]. Previous studies have suggested that the S protein of SARS-CoV experienced positive selection during SARS epidemic [20-22]. However, these studies did not find or did not analyze for positive selection among the replicase or accessory proteins, which may be equally important for SARS-CoV's adaptation to a new host. In order to systematically investigate the adaptive evolutionary process of SARS-CoVs, we employed the branch-site model to analyze the selective pressures that may act upon some key SARS-CoV functional proteins involved in virus entry, replication and assembly. Our results suggest that diversified selective forces act upon different proteins and during different epidemic phases.

## Methods

### Sequence data

A total of 156 sequences of SARS-CoVs or bat SARS-like-CoVs were retrieved from GenBank (129 complete genomes and 27 partial genomes) (see additional file 1). Based on these sequences, three datasets were constructed. Dataset 1 contains all Spike genes. Dataset 2 is a merged dataset that includes sequences of 4 main replicase domains of SARS-CoV: papain-like protease (PLpro), 3C-like protease (3CLpro), RNA dependent RNA polymerase (RdRp) and Helicase (Hel). Dataset 3 is a merged dataset that includes sequences of 7 ORFs: ORF3a, E, M, ORF6, ORF7a, ORF7b and N genes.

These protein-coding sequences are aligned based on translated protein sequences using Clustal W program implemented in BioEdit [23,24]. Prior to analysis all sequences that were identical to another within the dataset were removed, since previous studies have shown to have little effect on the detection of positive selection and contribute little evolutionary information [25]. Alignment gaps were manually removed based on the reference sequence of 31-HP03L_Tor2 (NC_004718).

The final composition of each dataset is as follows: dataset 1 contains 3765 bp of 59 S gene sequences; dataset 2 includes 35 sequences of replicase domains, 6435 bp in total (945 bp for PLpro, 918 bp for 3CLpro, 2769 bp for RdRp, 1803 bp for Hel) [17,26-28]; and dataset 3 contains 56 combined sequences, 3666 bp in total (822 bp for ORF3a, 228 bp for E, 663 bp for M, 189 bp for ORF6, 366 bp for ORF7a, 132 bp for ORF7b and 1266 bp for N).

### Phylogenetic analysis and reclassification of SARS-CoVs

**Figure 2:**
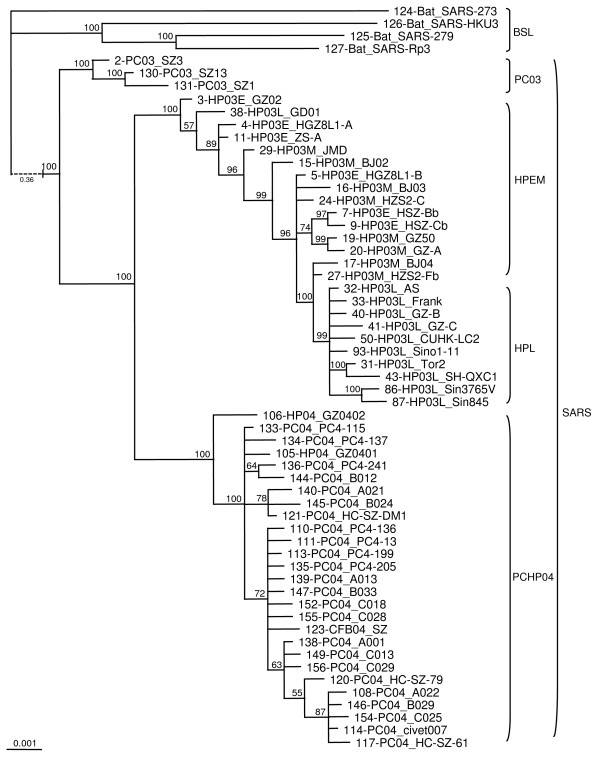
**Phylogenetic relationships of 59 S gene sequences of SARS-CoVs from human and animals**. The tree was generated with MrBayes 3.1.2 program. Posterior probabilities are shown on the nodes of the tree. Branch between BSL group and others was depicted with dotted line, because the branch was too long to be displayed at same scale. Bar, 0.001 nucleotide substitutions per site.

**Figure 3:**
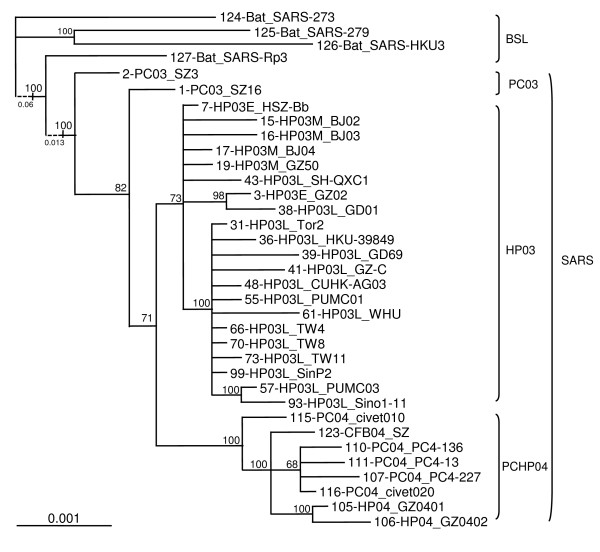
**Phylogenetic relationships of 35 replicase domains of SARS-CoVs from human and animals**. The tree was generated with MrBayes 3.1.2 program. Posterior probabilities are shown on the nodes of the tree. Bar, 0.001 nucleotide substitutions per site.

**Figure 4:**
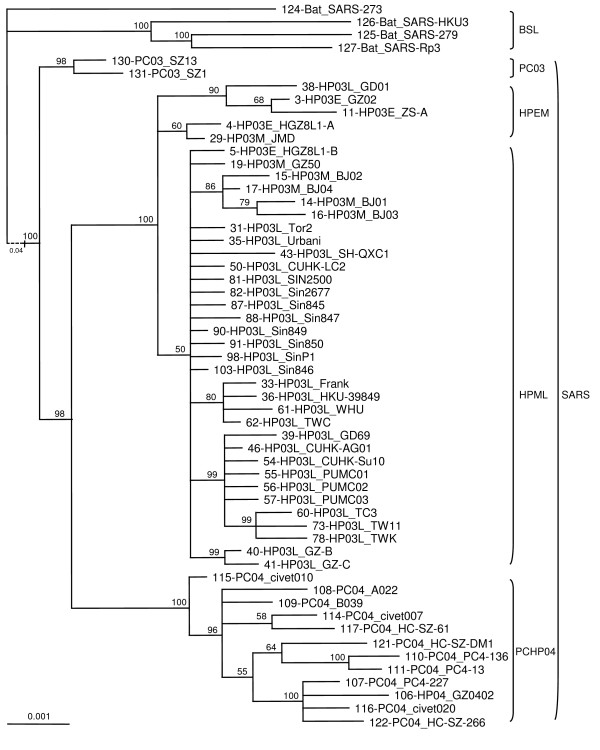
**Phylogenetic relationships of 56 3'-end ORF sequences of SARS-CoVs from human and animals**. The tree was generated with MrBayes 3.1.2 program. Posterior probabilities are shown on the nodes of the tree. Bar, 0.001 nucleotide substitutions per site.

For each dataset, a phylogenetic tree was built with MrBayes 3.1.2 (1,000,000 generations, sampled every 100 generations, burnin = 500, 4 chains) [29]. The tree topologies presented in figures 2, 3, 4 were used for different models. In previous studies, SARS-CoV isolates have been divided into five groups: 02–03 palm civets, 02–03 early, middle, late human patients, and 03–04 civet and human [20,21]. In the current study, we included an additional group containing the bat SARS-like-CoVs. Based on tree topologies and epidemiological information, we reclassified each dataset, such as to enable us to realistically investigate the adaptive evolution of SARS-CoVs in different hosts and during different epidemic periods. As showed in figures 2, 3, 4, the following groups were established: the BSL group, representing bat SARS-like-CoVs; the PC03 group, representing isolates from palm civets in 2003; the HPEM group, representing human patient isolates during early and middle epidemic phases in 2002–03; the HPL group, representing human patient isolates during late epidemic phase in 2003; the PCHP04 group, representing civet and human sequences from the 2003–04 epidemic phase; the HP03 group, representing all isolates collected from human patients during the epidemic period of 2002–03; and the HPML group, representing human patient isolates collected during the middle and late epidemic phases in 2003; and finally, the SARS group, representing all isolates collected from civets and human patients in 2002–04.

### Detection of recombination and positive selection

Since recombination can influence the detection of positive selection, we first tested for recombination in our data sets by using a genetic algorithm for recombination detection (GARD) [30]. Identified breakpoints by GARD were then assessed for significance by using Kishino-Hasegawa (KH) test in HYPHY package [31]. Since most sequences in SARS group have high similarity and increasing the number of sequences may dilute the signal, for each dataset, we choose 10 sequences for GARD analysis (four from BSL group: 124-Bat_SARS-273, 125-Bat_SARS-279, 126-Bat_SARS-HKU3, 127-Bat_SARS-Rp3; six from SARS group: 3-HP03E_GZ02, 15-HP03M_BJ02, 31-HP03L_Tor2, 106-HP04_GZ0402, 110-PC04_PC4-136, 130-PC03_SZ13).

To test for diversifying selection and to infer codon sites under positive selection, we mainly used CODEML program in the PAML 4.1 software package, which is based on the maximum likelihood algorithm of Yang and coworkers [32]. Three kinds of models (branch-specific, site-specific and branch-site) were employed to detect selective pressure among different branches and at different sites. The likelihood ratio test (LRT) was used to investigate whether the null hypothesis, where no positive selection is allowed, can be rejected against the alternative hypothesis, where positive selection is allowed [32]. The one ratio model (M0) assumes the same ω ratio for all branches and sites in the phylogeny. The free-ratio (FR) model assumes an independent ω ratio for each branch in the phylogeny. M0 and FR can be compared using LRT to examine whether ω ratios are different among lineages. The discrete model (M3) estimates ω for three classes of codon. Comparing M0 and M3 can test the variability of selective pressure among sites. When evidence for positive selection (ω > 1) was detected, the naïve empirical Bayes (NEB) method was used to calculate posterior probabilities for site classes. A higher posterior probability suggests strong support for a site to be under positive selection. In brief, the branch-specific model assumes variation among branches, but not among sites; the site-specific model assumes variation of selective pressure among sites, but not among branches. Both models are widely used to investigate selective pressure. However, if adaptive evolution occurs at a few time points and affects a few amino acids, these two models might lack power in detecting positive selection. To overcome this limitation, we also used branch-site model, which assumes that the ω ratio varies both among sites and among lineages [33,34]. In the branch-site model A (model A), the lineages of interest are set to be foreground, and the other lineages to be background. Selective constrains are assumed to vary across sites both along foreground and background, and a small fraction of sites only vary along foreground lineages. There are 3 ω ratios for foreground (0 < ω_0 _< 1, ω_1 _= 1, ω_2 _> 1) and 2 ω ratios for background (0 < ω_0 _< 1, ω_1 _= 1) in branch-site model A. When evidence for positive selection (ω > 1) was detected, both naïve empirical Bayes and Bayes empirical Bayes (BEB) were used to calculate posterior probabilities for site classes. Since NEB does not account for sampling errors, we used the BEB outputs as suggested by Yang [35]. The null model (model A') is same as model A, but ω_2 _= 1 is fixed. Branch-site model tends to be the most powerful of the three tests. In order to investigate the variation of selective pressure in different epidemic periods and hosts, we set each group of SARS-CoVs as foreground in turn to implement branch-site model. However, in such multiple tests, the probability of false rejection of at least one null hypothesis can be high. So we used Bonferroni correction to control the false positive rate, as it has been shown to be powerful when applied to the branch-site test [36]. As to dataset 1 and 3, we applied branch-site model to 6 groups on the tree, and for dataset 2, we applied branch-site model to 5 groups. So we used 0.0083 as the significance level for each branch-site tests in dataset 1 and 3, used 0.01 as the significance level for dataset 2. As indicated previously by Yang [33], these models sometimes fail to converge to maximum likelihood estimates. We thus performed each analysis at least twice using different starting values. Only identical data produced from both runs were considered reliable. All data are available upon request.

In order to examine the robustness of those positive selections identified by PAML, we also analyzed our datasets using HYPHY package accessed through the Datamonkey facility [37]. Datamonkey includes three methods for detecting sites under selection: single likelihood ancestor counting (SLAC), fixed effects likelihood (FEL) and random effects likelihood (REL). REL method is often the only method that can infer selection from small (5–15 sequences) or low divergence alignments and tends to be the most powerful of the three tests. So this method was run using the GTR substitution model on a neighbor-joining phylogenetic tree by the Datamonkey web server. In order to investigate selective pressure among different hosts and epidemic phases, we split each dataset (S protein, replicase domains, 3'-end ORFs) into appropriate groups for analysis.

## Results

### Phylogenetic analysis

For all genes analyzed, represented by S, replicase and 3'-end ORFs gene trees, at least four groups are apparent: BSL, PC03, HP03, PCHP04. As to the HP03 group, it can be subgrouped into HPEM and HPL in S gene tree, and HPEM and HPML in 3'-end ORF tree. It should be noted that the posterior probabilities for several nodes are low and there are some polytomies. These uncertainties could be due to some sequences in SARS group have high amino acid similarity, especially for replicase and 3'-end ORFs which are more conservative. However, previous studies suggested that the LRTs and qualitative results of ML parameter estimation were rather insensitive to tree topology [35,38-42]. For branch-site model, Bayesian site identification might be affected by tree topology [40]. Remarkably, one isolate (38-HP03L_GD01), which was isolated in the later epidemic phase in 2003, always clustered with the early phase isolates. A possible explanation could be that this patient was infected in the early epidemic phase, which is supported by sequence analysis; this isolate has 29 extra nucleotides between ORF8a and ORF8b, a feature shared among isolates from civets and early phase patients. Another isolate (5-HP03E_HGZ8L1-B), which was isolated in the early epidemic phase, tends to cluster with the middle phase isolates (Fig 2 and 4). This virus may be a transitional virus because it does not have the extra 29 nucleotides like the middle phase isolates.

### Detection of recombination

As showed in table 1 and additional file 2, GARD detected 9 breakpoints in dataset 1, and KH test indicated that 1 breakpoint (2301) was significant at *p*-value < 0.01 level. For dataset 2 and 3, GARD detected 2 and 5 breakpoints respectively, but none of them was significant after KH test.

**Table 1:** KH tests verify the significance of breakpoints estimated by GARD analysis

				*p-value*	
				
Dataset	Number of breakpoints	AIC_c _improvement	Breakpoint location	LHS	RHS
Spike	9	588.485	776	1.000	0.220
			933	0.059	0.427
			1257	1.000	0.464
			1485	1.000	1.000
			2067	1.000	0.670
			**2301**	**0.002**	**0.002**
			2592	0.893	0.061
			2916	0.085	0.988
			3501	1.000	0.659
Replicase	2	30.414	1230	1.000	0.507
			4398	1.000	0.040
3'-end ORFs	5	254.203	454	0.363	0.306
			729	0.210	0.001
			1091	0.010	0.078
			1927	0.254	0.005
			3321	1.000	0.353

KH test was used in both directions to compare phylogenies constructed from the alignment segment to the left hand side (LHS) and right hand side (RHS) of each estimated breakpoint. All *p-values *have been adjusted by Bonferroni correction.

### Positive selection on the S protein of SARS-CoV

We analyzed the entire S gene of 156 isolates. Because several isolates were identical at the amino acid sequence level, we eliminated them from the dataset since previous analyses indicated that contribute limited evolutionary information [19,25]. Therefore, 59 sequences were compiled into dataset 1. Table 2 presents the analysis results of dataset 1. The analyses of branch-specific model (FR) indicate that selective pressure varied along branches. Many branches in the HPEM and PCHP04 groups clearly have higher ω ratios. The LRT statistic for comparing M0 and FR is significant, which confirm the heterogeneous selective pressure along branches. According to the site-specific model (M3), 1.3% sites among S protein are under positive selection with ω = 3.214. Furthermore, this model identifies 9 sites under positive selection at posterior probability p > 90% level (Table 2). All these sites are distributed within the S1 domain.

**Table 2:** Maximum likelihood (ML) estimates for 59 S genes of SARS-CoV

Models	d.f.	Parameters under null model	Parameters under alternative model	lnL_0_ (lnL_1_)	2Δ*l*	*P*-value	Positively selected sites*
Branch Model M0 vs. FR	114	M0 (one ratio) ω = 0.081	Free Ratio ω = 0~∞	-12834.110 (-12656.604)	355.006	< 0.001	Not allowed
Site Model M0 vs. M3	4	M0 (one ratio) ω = 0.081	M3 (discrete, *K *= 3) *p*_0 _= 0.732, ω_0 _= 0.015 *p*_1 _= 0.255, ω_1 _= 0.285 *p*_2 _= 0.013, **ω **_2_**= 3.214**	-12834.110 (-12616.008)	436.204	< 0.001	142,**311**, 430, **462, 479, 540** 609, **626, 665**
Branch-site model A							
BSL group as foreground MA' vs. MA	1	MA' (fix ω_2_= 1) *p*_0 _= 0.912, ω_0 _= 0.047 *p*_1 _= 0.088 (*p*_2a_+*p*_2b _= 0)	MA *p*_0 _= 0.912, ω_0 _= 0.047 *p*_1 _= 0.088, ω_2 _= 1.000 (*p*_2a_+*p*_2b _= 0)	-12661.687 (-12661.687)	0	1.000	None
PC03 group as foreground MA' vs. MA	1	MA' (fix ω_2_= 1) *p*_0 _= 0.707, ω_0 _= 0.046 *p*_1 _= 0.069 (*p*_2a_+*p*_2b _= 0.224)	MA *p*_0 _= 0.782, ω_0 _= 0.046 *p*_1 _= 0.076, **ω **_2_**= 1.592** (*p*_2a_+*p*_2b _= 0.142)	-12658.258 (-12658.246)	0.024	0.877	None
HPEM group as foreground MA' vs. MA	1	MA' (fix ω_2_= 1) *p*_0 _= 0.587, ω_0 _= 0.044 *p*_1 _= 0.055 (*p*_2a_+*p*_2b _= 0.358)	MA *p*_0 _= 0.885, ω_0 _= 0.045 *p*_1 _= 0.083, **ω **_2_**= 28.756** (*p*_2a_+*p*_2b _= 0.032)	-12646.115 (-12638.329)	15.572	< 0.001	49, **75**, 344, 360, 501, **778** 794, 860, 861 1001, 1148, **1163** 1179, 124
HPL group as foreground MA' vs. MA	1	MA' (fix ω_2 _= 1) *p*_0_= 0.400, ω_0 _= 0.045 *p*_1_= 0.038 (*p*_2a_+*p*_2b _= 0.562)	MA *p*_0 _= 0.400, ω_0 _= 0.045 *p*_1_= 0.038, ω_2 _= 1.000 (*p*_2a_+*p*_2b _= 0.562)	-12650.732 (-12650.732)	0	1.000	
PCHP04 group as foreground MA' vs. MA	1	MA' (fix ω_2_= 1) *p*_0_= 0.718, ω_0 _= 0.045 *p*_1_= 0.057 (*p*_2a_+*p*_2b _= 0.225)	MA *p*_0_= 0.901, ω_0_= 0.045 *p*_1_= 0.072, **ω **_2_**= 57.205** (*p*_2a_+*p*_2b _= 0.027)	-12626,601 (-12569.700)	113.802	< 0.001	78, 91, **108**, 113, **147, 227,** **243, **425, **440,** **462, 479, 609**, **613,** 632, **743,** **765**, 839, 844, 856, 900, **1052** **1080**
SARS group as foreground MA' vs. MA	1	MA' (fix ω_2_= 1) *p*_0_= 0.753, ω_0 _= 0.024 *p*_1_= 0.035 (*p*_2a_+*p*_2b _= 0.212)	MA p_0_= 0.792, ω_0_= 0.027 *p*_1_= 0.034, **ω **_2_**= 1.989** (*p*_2a_+*p*_2b _= 0.174)	-12498.107 (-12488.470)	19.274	< 0.001	**2,** 7, 9, **12,** 14, **20,** **27, 33, 37, 43, 58, 68,** 70, **75, 84,** 107, **108,** 131, 134, **137,** 139 **147, 151,** 154, **163, 165,** **167, 169, 174, 199, 201,** **214, 227, 230, 237, 239** 242, **243, 244, 248,** 249, 294, **333, 336, 344, 353,** **391, 392, 426,** 431, **440, 442, 457,** 459, **462,** 479, **480,** 487, **488,** 494, 607, **613,** 644, **729, 732, 743,** 754, **758, 765, 778,** **1052, 1080,** 1148, **1163**

* Positively selected sites are identified with posterior probability p > 90%. In boldface, p > 95%.

The results of branch-site model revealed that no evidence of positive selection was found in the BSL, PC03 and HPL groups. For the HPEM group, the results indicated that 3.2% sites of S gene are subjected to strong positive selection with ω = 28.756. At p > 90% level, 14 specific sites were identified as potentially under positive selection (Table 2). For the PCHP04 group, 2.7% codon sites of the S gene are driven by strong positive selection with ω = 57.205. Twenty two positively selected sites were identified in this group (p > 90%). Fourteen of them are in S1 and eight in S2 domain. For the selection of entire SARS-CoVs from the two epidemics, the branch-site model A analyses indicated that 17.4% sites are under positive selection with ω = 1.989. A total of 74 sites were identified as potentially under positive selection along these lineages at 90% cutoff. In order to intuitively represent the distribution of these positively selected sites, we constructed the additional file 3, from which we can find that most of these sites distribute in S1 domain.

HYPHY package analysis accessed through Datamonkey facility also detected positive selection in HPEM and PCHP04 groups (with *d*_*N*_*-d*_*S *_= 0.061 and 0.938 respectively), but not in BSL, PC03 and HPL groups. As indicated in table 3, we also identified some positively selected sites, most of which are identical to those identified by the branch-site model A.

**Table 3:** REL analysis results for three datasets

**Groups**^a^	**No. of sequences^d^**	**Mean *d*_*N*_-*d*_*S*_^e^**	**Positively selected site(s)**
**Spike**			
BAT	4	-0.957	
PC03	3	-0.904	
HPEM	14	**0.061**	49, 75, 77, 144, 239, 244, 311, 344, 778, 860, 861, 1001, 1148, 1163, 1179, 1247
			
HPL	11	-0.138	
PCHP04	27	0.938	
SARS^b^	40	**0.361**	75, 147, 227, 239, 243, 244, 311, 462, 479, 609, 613, 743, 765, 778, 1080, 1163
**Replicase**			
BAT	4	-0.985	
HP03	21	**0.008**	654
PCHP04	8	-0.774	
SARS	31	-0.561	
			
**3'-end ORFs**			
BAT	4	-0.91	
HPEM	5	-0.42	
HPML	33	**0.152**	
PCHP04	12	-0.571	
SARS^c^	40	-0.301	

a. At least 3 sequences are needed for REL analysis, so PC03 groups of dataset 2 and 3 were not analyzed.

b. The upper limit in number of sequences for REL test is 40, so 15 sequences were removed from original SARS group (removed sequences' number: 33, 40, 43, 50, 86, 108, 110, 111, 123, 135, 139, 144, 147, 152, 156)

c. 12 sequences were removed from original SARS group (removed sequences' number: 5, 19, 50, 56, 57, 81, 82, 88, 91, 103, 107, 111)

d. As a rule of thumb, at least 10 sequences are needed to detect selection at a signal site with reliability. So some of the results may be not reliable because of not enough sequences are available for some groups.

e. Because *d*_*S *_could be 0 for some sites, Datamonkey reports *d*_*N*_-*d*_*S *_in place of *d*_*N*_/*d*_*S*_

### Positive selection on replicase domains of SARS-CoV

PLpro, 3CLpro, RdRp and Hel are the major domains for coronavirus replication [43,44]. We merged these four domains into one supergene for analysis because: 1) Yang et al. reported that gene concatenating analysis produced same outcomes as those obtained from analysis of separate genes [42]; 2) separate analysis results in mechanical repeats; 3) concatenating analysis can provide additional information because of additional number of sequences for the merged dataset, compared to separate dataset. Therefore, dataset 2 consists of 35 concatenated sequences from 129 complete genomes.

As presented in table 4, the results of branch model analysis reveal that the ω ratio varies from 0 to infinite along different branches. This implies that selective pressures among these domains vary in different hosts and at different epidemic phases, though these domains are the most conserved regions in CoV. Analysis using the discrete model (M3) detected no sign of positive selection in the dataset 2, although it suggests that the ω ratios vary significantly among different amino acid sites as indicated by LRT.

**Table 4:** Maximum likelihood (ML) estimates for 35 merged replicase genes of SARS-CoV

Models	d.f.	Parameters under null model	Parameters under alternative model	lnL_0_ (lnL_1_)	2Δ*l*	*P*-value	Positively selected sites*
Branch Model M0 vs. FR	66	M0 (one ratio) ω = 0.024	Free Ratio ω = 0~∞	-14460.634 (-14354.549)	212.17	< 0.001	Not allowed
Site Model M0 vs. M3	4	M0 (one ratio) ω = 0.024	M3 (discrete, *K *= 3) *p*_0_= 0, ω_0_= 0 *p*_1_= 0.972, ω_1_= 0.016 (*p*_2_= 0.028), ω_2 _= 0.360	-14460.634 (-14447.201)	26.866	< 0.001	None
Branch-site model A							
BSL group as foreground MA' vs. MA	1	MA' (fix ω_2_= 1) *p*_0 _= 0.993, ω_0 _= 0.020 *p*_1 _= 0.007 (*p*_2a_+*p*_2b _= 0)	MA *p*_0 _= 0.993, ω_0 _= 0.020 *p*_1 _= 0.007, ω_2 _= 1.000 (*p*_2a_+*p*_2b _= 0)	-14449.205 (-14449. 205)	0	1.000	None
PC03 group as foreground MA' vs. MA	1	MA' (fix ω_2 _= 1) *p*_0 _= 0.993, ω_0 _= 0.020 *p*_1 _= 0.007 (*p*_2a_+*p*_2b _= 0)	MA *p*_0 _= 0.993, ω_0 _= 0.020 *p*_1 _= 0.007, ω_2 _= 1.000 (*p*_2a_+*p*_2b _= 0)	-14449. 205 (-14449. 205)	0	1.000	None
HP03 group as foreground MA' vs. MA	1	MA' (fix ω_2_= 1) *p*_0 _= 0.322, ω_0 _= 0.015 *p*_1 _= 0.002 (*p*_2a_+*p*_2b _= 0.676)	MA *p*_0_= 0.913, ω_0_= 0.015 *p*_1_= 0.006, **ω **_2_**= 11.093** (*p*_2a_+*p*_2b _= 0.081)	-14389.596 (-14386.122)	6.948	0.008	23, **123**, 222, 236, 237, 250, 266, 375, 377, 409, 504, 563, 646, **654,** 884, 1234, 1259, 1482, 1491, 1786, 1866, 1869, 1878, 1963, 1995, 2010, 2032, 2034
PCHP04 group as foreground MA' vs. MA	1	MA' (fix ω_2_= 1) *p*_0_= 0.761, ω_0_= 0.018 *p*_1_= 0.006 (*p*_2a_+*p*_2b _= 0.234)	MA *p*_0_= 0.760, ω_0_= 0.018 *p*_1_= 0.006, ω_2 _= 1.000 (*p*_2a_+*p*_2b _= 0.234)	-14435.921 (-14435.921)	0	1.000	None
SARS group as foreground MA' vs. MA	1	MA' (fix ω_2_= 1) *p*_0 _= 0.850, ω_0 _= 0.012 *p*_1 _= 0.005 (*p*_2a_+*p*_2b _= 0.145)	MA *p*_0 _= 0.857, ω_0 _= 0.012 *p*_1 _= 0.005, ω_2_= 1.061 (*p*_2a_+*p*_2b _= 0.138)	-14405.997 (-14405.994)	0.006	0.938	

* Positively selected sites are identified with posterior probability p > 90%. In boldface, p > 95%.

Utilizing the branch-site model A analysis indicated that there is no positive selection in the BSL, PC03 and PCHP04 groups. However, the model A analysis revealed that among HP03 group about 8.1% codon sites of these 4 domains are potentially under strong positive selection with ω = 11.093 and 28 sites were identified (7 in PLpro, 5 in 3CLpro, 7 in RdRp, 9 in HEL). Weak positive selection (*d*_*N*_*-d*_*S *_= 0.001) was also detected from HP03 group by using HYPHY package but not other groups (Table 3).

### Positive selection on 3'-end ORFs of SARS-CoV

The 3'-end of SARS-CoV genome encodes 11 ORFs: ORF3a, ORF3b, ORF4 (E), ORF5 (M), ORF6, ORF7a, ORF7b, ORF8a, ORF8b, ORF9a (N), and ORF9b. The E, M, N proteins are structural proteins of SARS-CoV and the other proteins are accessory proteins. Because the coding regions of ORF3b and ORF9b overlap partially or completely with those of ORF3a and N, we excluded the ORF3b and the ORF9b from this analysis. The ORF8a and ORF8b are present as two separate ORFs in most human isolates but as a single ORF (ORF8ab) in isolates from animals and early phase human due to the presence of extra 29 nt in this region, thus resulting in the fusion of ORF8a and ORF8b. Because of the difficulty in obtaining a reliable alignment in this region, ORF8 (a, b or ab) was excluded from our analysis as well. For similar reasons as mentioned above, we merged the 7 remaining ORFs into a supergene for analysis.

As presented in table 5, the results of FR model analysis revealed that selective pressures vary among lineages. The results of M3 model also implied variation in selective pressure among different amino acid sites. However, the M3 model did not detect any sign of positive selection. The results of branch-site model A revealed that, except for the BSL, PC03 and HPEM groups, the other groups displayed positive selection signatures (Table 5). For the HPML group, about 12.2% sites of these ORFs were shown to be under positive selection with ω = 9.863. Twenty five specific sites were identified: 6 in ORF3a (11, 29, 85 129, 136, 222); 4 in E (279, 280, 304, 319); 9 in M (377, 388, 418, 423, 436, 449, 463, 469, 504); 1 in ORF6 (584); 1 in ORF7a (696); and 4 in N (850, 932, 954, 993). When the PCHP04 group was defined as foreground, the branch-site model A analysis estimated that 1.9% sites were under positive selection with ω = 22.447 and four sites were identified to be under positive selection (2 in ORF3a, 1 in ORF6, 1 in N). For the whole SARS-CoV collection, the branch-site model A analysis revealed 12.2% sites of these ORFs to be under positive selection with ω = 3.138. A total of 17 sites were identified at p > 90% level. Among them, 9 are located in ORF3a, 3 in M, 2 in ORF6 and 3 in N. In addition, a large number of sites were identified to be potentially under positive selection at p > 70% level (see additional file 3B).

**Table 5:** Maximum likelihood (ML) estimates for 56 merged 3'-end ORFs of SARS-CoV

Models	d.f.	Parameters under null model	Parameters under alternative model	lnL_0_ (lnL_1_)	2Δ*l*	*P*-value	Positively selected sites*
Branch Model M0 vs. FR	108	M0 (one ratio) ω = 0.170	Free Ratio ω = 0~∞	-9142.692 (-9055.881)	173.623	< 0.001	Not allowed
Site Model M0 vs. M3	4	M0 (one ratio) ω = 0.170	M3 (discrete, *K *= 3) *p*_0_= 0, ω_0_= 0 *p*_1_= 0.866, ω_1_= 0.058 (*p*_2_= 0.134),ω_2_= 0.986	-9142.692 (-9093.135)	99.114	< 0.001	None
Branch-site model A							
BSL group as foreground MA' vs. MA	1	MA' (fix ω_2_= 1) *p*_0_= 0.868, ω_0_= 0.059 *p*_1_= 0.132 (*p*_2a_+*p*_2b _= 0)	MA *p*_0_= 0.868, ω_0_= 0.059 *p*_1_= 0.132, ω_2_= 1.000 (*p*_2a_+*p*_2b _= 0)	-9093.137 (-9093.137)	0	1.000	None
PC03 group as foreground MA' vs. MA	1	MA' (fix ω_2_= 1) *p*_0_= 0.868, ω_0_= 0.059 *p*_1_= 0.132 (*p*_2a_+*p*_2b _= 0)	MA *p*_0_= 0.868, ω_0_= 0.059 *p*_1_= 0.132, ω_2 _= 1.000 (*p*_2a_+*p*_2b _= 0)	-9093.137 (-9093.137)	0	1.000	None
HPEM group as foreground MA' vs. MA	1	MA' (fix ω_2_= 1) *p*_0_= 0.855, ω_0_= 0.059 *p*_1_= 0.130 (*p*_2a_+*p*_2b _= 0.015)	MA *p*_0_= 0.861, ω_0_= 0.059 *p*_1_= 0.131, **ω **_2_**= 4.300** (*p*_2a_+*p*_2b _= 0.008)	-9093.127 (-9093.088)	0.078	0.780	
HPML group as foreground MA' vs. MA	1	MA' (fix ω_2_= 1) *p*_0_= 0.125, ω_0_= 0.047 *p*_1_= 0.017 (*p*_2a_+*p*_2b _= 0.858)	MA *p*_0_= 0.772, ω_0_= 0.046 *p*_1_= 0.106, **ω **_2_**= 9.863** (*p*_2a_+*p*_2b _= 0.122)	-9069.427 (-9065.120)	8.614	0.003	**11, 29**, 85, 129, 136, **222, 279,** 280, **304, 319,** 377, 388, **418, 423, 436,** 449, 463, 469, 504, 584, **696, 850, 932,** 954, **993**
PCHP04 group as foreground MA' vs. MA	1	MA' (fix ω_2_= 1) *p*_0_= 0.690, ω_0_= 0.055 *p*_1_= 0.097 (*p*_2a_+*p*_2b _= 0.213)	MA *p*_0_= 0.862, ω_0 _= 0.057 *p*_1_= 0.119, **ω **_2_**= 22.447** (*p*_2a_+*p*_2b _= 0.019)	-9087.427 (-9076.176)	22.502	< 0.001	**25, 259, 609, 1184**
SARS group as foreground MA' vs. MA	1	MA' (fix ω_2 _= 1) *p*_0 _= 0.664, ω_0_= 0.033 *p*_1_= 0.066 (*p*_2a_+*p*_2b _= 0.270)	MA *p*_0_= 0.804, ω_0_= 0.037 *p*_1_= 0.074, **ω **_2_**= 3.138** (*p*_2a_+*p*_2b _= 0.122)	-9058.932 (-9051.231)	15.402	< 0.001	**11, 15, 81**,**117, 120, 121, ** **171,** 193, **259, 355, 361, 560,** ** 609, 628, 830, 850, 1184**

* Positively selected sites were identified with posterior probability p > 90%. In boldface, p > 95%.

## Discussion

Natural selection generally leads to a reduction in deleterious mutations while promoting advantageous mutations. If a gene is highly divergent, there are two main explanations: first, it may be due to high mutation rate or relaxed selective constraint, in which case the gene may be free to mutate mainly because it has no fitness or function; or second, due to positive selection which is promoted by natural selection and the gene usually has highly important functions [45]. Virus entry, replication, assembly and release are the main steps of viral life cycle. Proteins involved in each of these steps may undergo adaptive evolution after a virus invades a new host.

Recombination and mutation are two important evolutionary mechanisms driving gene diversity and adaptation. Since recombination can affect the detection of positive selection, we first tested for recombination in our datasets [46]. GARD detected no evidence of recombination within the replicase and 3'-end ORFs, while one putative breakpoint in spike protein was detected. Whether there is any recombination among SARS-CoV is still debatable [13,22,47-49]. Previous studies suggested putative recombination only when analysis of SARS-CoV sequences were put together with other coronaviruses [47-49]; however, when analyses were focused solely on SARS-CoV sequences, recombination could not be detected [13,22]. We also tested recombination in SARS group alone for each dataset. No evidence of recombination was detected by GARD. These results might imply that there could be some ancient recombination events occurred between bat SARS-like-CoV and the ancestor of SARS-CoV, which drove the bat SARS-like-CoV adaption to civet and human. Nevertheless, previous studies had revealed that detection of positive selection by LRT method was robust to low levels of recombination (with fewer than three recombination events), and identification of sites under positive selection by the empirical Bayes method appeared to be less affected than the LRT by recombination [46]. Overall, the issue of recombination among RNA viruses is highly controversial because the putative recombination events described were detected only by utilizing computationally-demanding phylogenetic analyses (split decomposition and/or maximum likelihood methods). Therefore, caution should be used when inferring conclusions about putative recombination events that are based only on such analyses. Because viable clonal recombinant viruses have been rarely observed in nature, for natural recombination leading to the transmission of a recombinant strain to be conclusively confirmed, the following prerequisites should be met: (i) the recombinant crossover should be demonstrated in a single PCR amplicon following cloning to ensure it occurs in a single DNA molecule; (ii) the recombination should be demonstrated repeatedly in clonal populations of viable virus (e.g. a plaque harvest or limited endpoint dilution; and (iii) the recombinant should maintain adequate sequence conservation during post-recombination evolution [50].

The S protein is a structural protein of coronavirus. It has a crucial role in the binding of virus to host receptor and subsequent fusion between the viral and host membranes, both processes being important for virus entry into host cell. In the case of several mammalian and avian coronaviruses, the S protein is cleaved into S1 and S2. The former contains receptor attachment sites and the later is involved in the fusion of CoV onto host cell. The S1 subunit, which usually has high divergence, contains a receptor binding domain (RBD); the S2 subunit, which is comparatively more conserved, contains two heptad repeat (HR) domains [51]. Several studies revealed that the S gene of SARS experienced noticeable positive selection during the SARS epidemics, especially in the early and middle phases [20-22]. However, our analyses indicated that the S protein of SARS-CoV underwent a stepwise adaptive process subsequent to its spillover into the civet and human populations. In the BSL group, our analyses suggest that the bat SARS-like-CoVs experienced purifying selection, indicating that the S gene is relatively stable in bats. In palm civet, SARS-CoV experienced strong positive selection as indicated by the results of PCHP04 group. The failure to identify significant positive selection in PC03 group was most likely due to the limited number of sequences available for analyses (only four sequences for PC03 group and two of them have identical S gene sequences). During the early and middle phases of the 2002–2003 SARS epidemic in human population, a small fraction of sites among the S protein were under strong positive selection. In contrast, isolates from the late phase showed no sign of positive selection, implying the S protein became stable again after two stages of adaptive evolution. Our analysis using the HYPHY package accessed through Datamonkey facility also revealed similar evolutionary patterns for the S protein (Table 3). Taken together, these results support the hypothesis that SARS-CoVs originated in bats, that the spill over into civets and humans were recent events and that the two SARS epidemics that took place one year apart, were results of independent animal-to-human transmissions [7,20]. The major sequence difference of the S genes between bat SARS-like-CoVs and civet/human SARS-CoVs suggests that there may be other more closely related SARS-CoVs in bats or there may be other unknown intermediate animal host(s) in the transmission of the virus(es) from bats to civets [49].

Among the SARS-CoVs from human and palm civets, numerous sites are identified to be potentially under positive selection (Table 2 and see additional file 3A). Those sites inferred from different SARS epidemic phases reflect the adaptation process of SARS-CoVs. Those sites identified from the PCHP04 group may be important for SARS-CoV adaptation to palm civets. Those sites identified from the HPEM group may be important for SARS-CoV adaptation to human. Most of these sites, especially from the entire SARS group, are located in the S1 domain that contains the receptor binding domain. Zhang et al. [22] previously identified 12 positively selected sites in the SARS-CoV group, all of which were confirmed in our current study. The greater number of sites identified in our study is likely due to the fact that the branch-site model is more powerful than the site-specific model. Some of these sites have been confirmed by experimental data to be crucial for the adaption of SARS-CoVs to human. For example, it has been found that adaptation of S protein to human angiotensin converting enzyme 2 (ACE2) is facilitated by alteration of residue 479 to asparagine and of 487 to threonine [52,53]. Also, using site-directed mutagenesis, Zhu and Chakraborti identified that residues 344, 392, 426, 431, 479, 480, 487, 488 and 494 are important for the binding of RBD with ACE2 and SARS-CoV antibody [54,55]. In RBD, there are eight newly identified sites (333, 336, 353, 391, 440, 442, 457, 459, and 462) which have not been proved to be critical for RBD and ACE2 interaction. Furthermore, there are ~60 sites to be under positive selection beside RBD. Although evaluation of every observed site under positive selection by reverse genetics would not be realistic or feasible, generation and evaluation of mutant viruses based on sites located within or adjacent to functional domains could provide clues for the genetic aetiology of SARS adaptation to new hosts and emergence.

The first two thirds of coronavirus genome encode two large polyproteins: P1a and P1ab, which are cleaved by virus-encoded proteinases (PLpro and 3CLpro) into 16 non-structure proteins (nsp1–nsp16) playing important role during coronavirus replication. Because the P1ab is too big (~21 kb), we analyzed four most important domains related to viral replication: PLpro, 3CLpro, RdRp and Hel, which correspond to nsp3, nsp5, nsp12 and nsp13, respectively [17,43,44]. Our results revealed that, unlike the adaptive evolutionary pattern of S protein, these replicase domains did not experience positive selection in bat and palm civet, but underwent strong positive selection in human patient. Moreover the evidence of positive selection is stronger in the later phases than that observed in the early and middle phases (data no shown). Furthermore, our analysis using HYPHY package observed very weak positive selection in the HP03 group but not among the other groups (Table 3). These results to some extent differ from the observations derived from several previous studies. Using pairwise analysis of the *d*_N_/*d*_S_, Song *et al*. found that the average ω ratio of S protein for the early phase was larger than that for the middle phase, which in turn was larger than the ratio for the late phase. A similar pattern was found in ORF1a and ORF3a but not in ORF1b and nsp3, which were suggested under purifying selection during the whole course of the epidemic. They identified over 200 single-nucleotide variations (SNVs) and inferred the importance of some SNVs on SARS-CoV adaptive evolution [21]. Zhang *et al*. also investigated the adaptive evolution of the S protein employing the site specific model, but they did not observe any positive selection in RdRp, Hel or nsp3 [21,22]. The best explanation for this apparent discrepancy is that the methods used in their studies were more conservative than the branch-site model used in our study, and hence were not able to detect positively selected changes among the highly conserved genes. Alternatively, it may be due to the use of concatenating analysis in our study, which can provide additional information due to the compiling of more dissimilar sequences for datasets. For the replicase genes of bat and civet isolates, there was no sign of positive selection. This is probably due to the following reasons: 1) the civet isolates were collected within a very short time period and thus there was not enough time for adaptive evolution; 2) civet cells are very suitable for SARS-CoV replication which may imply that the civet is a perfect intermediate host for SARS-CoV; and 3) the bat isolates might have completely adapted to their hosts and hence were under no further selective pressure for evolution.

As to the 3'-end ORFs, the most diversifying selection happened in the middle and late phases of the SARS epidemic in 2003–2004. No positive selection was found in the BSL, PC03 and HPEM groups. When the isolates from two epidemics of 2003 and 2004 were investigated together, 12.2% sites in these 7 ORFs were shown to be under positive selection (Table 5). In addition to a few sites identified at p > 90% level, many sites are inferred to be under positive selection at 70–80% posterior probability (see additional file 3B). Most of these sites are located in ORF3a, E, M and ORF6, implying these four genes may play a more important role for SARS-CoV adaptation in a new host. Our results based on the REL method showed weak positive selection in HPML group, but not in other groups (Table 3). The failure to identify specific sites under positive selection could be due to weak and thus undetected positive selection. These results suggested that the 3'end-ORFs underwent positive selection after SARS-CoV spilled out into civet and human populations, and adaptive evolution mainly happened in the middle and later phases in 2003. Song *et al*. previously suggested that the 3a protein evolved adaptively as S protein [21]. By estimating mutation rates, Zhao *et al*. suggested that the non-synonymous substitution rates were comparatively high in E, M and N [56]. ORF3a encodes a protein of 274 amino acids. A recent study indicated that the 3a protein forms a potassium sensitive channel that may promote virus release and may be important for modulating expression of S on the cell surface [16,57]. 3a protein also interacts with the structural proteins S, E, M [57,58]. Therefore, amino acid changes in 3a protein might be necessary to maintain the interaction between 3a and other proteins. E and M protein play a pivotal role in viral morphogenesis, assembly and budding. Co-expression of E and M was shown to produce virus-like particles, roughly the same size and shape as virions [59]. N protein is important for viral packaging which is the first step in the assembly of infectious SARS viruses [60]. Thus the amino acid changes in these three structural proteins may be critical for virus assembly in the new host. SARS-CoV ORF 6 protein can enhance the virulence of attenuated murine coronavirus (MHV) [61], as well as stimulate cellular DNA synthesis [62]. A recent study showed that ORF 6 protein inhibited both interferon synthesis and signaling [63]. These findings suggested that ORF 6 may have a role in enhancing virus replication or assembly. ORF 7a protein inhibits cellular protein synthesis and blocks cell cycle progression at G0/G1 phase, suggesting that 7a may play important roles in the life cycle of SARS-CoV and the pathogenesis induced by SARS-CoV [64,65]. The function of other accessory proteins remains to be determined. Overall, these findings suggest that the 3'-end ORFs play important roles for SARS-CoV replication, assembly and release. Collectively, amino acid changes in these proteins could play a role in modulating the host switch of SARS-CoV.

## Conclusion

We systematically analyzed the individual SARS-CoV proteins important for virus entry, replication and assembly. The results suggested that SARS-CoVs experienced a stepwise adaptation to humans. In palm civets and humans during the early and middle epidemic phases, virus entry-mediating protein S experienced strong positive selection. In contrast, the replicase proteins experienced positive selection only in human patients but not in palm civets, implying that the palm civet is a suitable intermediate host for SARS-CoV replication. The proteins involved in virus assembly and release mainly underwent positive selection during the middle and later epidemic phases. These results highlight the spectacular dynamics of SARS-CoV evolution in a narrow time window, period of epidemic, support the zoonotic origin of SARS and suggest that some amino acid sites may be critical for viral adaptation in different hosts. Collectively, these results suggest that the development of SARS-CoV reverse genetics system will facilitate further molecular and/or epidemiological investigations to elucidate role of adaptive virus evolution in future emergence events.

## Authors' contributions

XT carried out the data collection, analysis, and wrote the manuscript. GL and YZhang participated in the data analysis. NV participated in the data analysis and manuscript revising. ZS, YZhong, LFW and SZ participated in its design and revised the manuscript. SZ supervised and coordinated the project. All authors read and approved the final manuscript.

## Supplementary Material

Additional file 1 **Table S1.** List of 156 sequences of SARS-CoV analyzed in this study. Click here for file

Additional file 2 **Figure S1.** Detection of recombination with GARD method. (A) putative breakpoints in spike gene; (B) putative breakpoints in replicase domains; (C) putative breakpoints in 3'-end ORFs. Click here for file

Additional file 3 **Figure S2.** The distribution of positively selected sites identified using the branch-site model A (SARS group as foreground). (A) Positively selected sites among S protein of SARS-CoV; (B) positively selected sites among 3'-end ORFs of SARS-CoV. The most significant peaks (p >95%) were colored in red. Click here for file
