# Supplementary material for: Differential stepwise evolution of SARS coronavirus functional proteins in different host species
Source: BMC Evol Biol. 2009 Mar 5;9:52. doi: 10.1186/1471-2148-9-52 (PMC2676248; doi:10.1186/1471-2148-9-52)
Supplement: Additional file 1 — Table S1. List of 156 sequences of SARS-CoV analyzed in this study. [file 1471-2148-9-52-S1.pdf]

Additional file 1 - Table S1. List of 156 sequences of SARS-CoV analyzed in this study.

Number	Accession Number	Name Used in this Paper	Place and Date of Sampling*/Submission#	Number	Accession Number	Name Used in this Paper	Place and Date of Sampling*/Submission#
Complete genome				79	AP006560	79-HP03L_TWS	Taiwan, 30 Jul 2003 #
1	AY304488	1-PC03_SZ16	Shenzhen, 07 May 2003 *	80	AP006561	80-HP03L_TWY	Taiwan, 30 Jul 2003 #
2	AY304486	2-PC03_SZ3	Shenzhen, 07 May 2003 *	81	AY283794	81-HP03L_SIN2500	Singapore, 27 Apr 2003 #
3	AY390556	3-HP03E_GZ02	Guangzhou, 11 Feb 2003 #	82	AY283795	82-HP03L_Sin2677	Singapore, 27 Apr 2003 #
4	AY394981	4-HP03E_HGZ8L1-A	Guangzhou, 19 Sep 2003 #	83	AY283796	83-HP03L_Sin2679	Singapore, 27 Apr 2003 #
5	AY394982	5-HP03E_HGZ8L1-B	Guangzhou, 19 Sep 2003 #	84	AY283797	84-HP03L_Sin2748	Singapore, 27 Apr 2003 #
6	AY394984	6-HP03E_HSZ-A	Guangdong, 19 Sep 2003 #	85	AY283798	85-HP03L_Sin2774	Singapore, 27 Apr 2003 #
7	AY394985	7-HP03E_HSZ-Bb	Guangdong, 19 Sep 2003 #	86	AY559084	86-HP03L_Sin3765V	Singapore, 24 Feb 2004 #
8	AY394994	8-HP03E_HSZ-Bc	Guangdong, 19 Sep 2003 #	87	AY559093	87-HP03L_Sin845	Singapore, 24 Feb 2004 #
9	AY394986	9-HP03E_HSZ-Cb	Guangdong, 19 Sep 2003 #	88	AY559095	88-HP03L_Sin847	Singapore, 24 Feb 2004 #
10	AY394995	10-HP03E_HSZ-Cc	Guangdong, 19 Sep 2003 #	89	AY559085	89-HP03L_Sin848	Singapore, 24 Feb 2004 #
11	AY394997	11-HP03E_ZS-A	Zhongshan, 19 Sep 2003 #	90	AY559086	90-HP03L_Sin849	Singapore, 24 Feb 2004 #
12	AY394996	12-HP03E_ZS-B	Zhongshan, 19 Sep 2003 #	91	AY559096	91-HP03L_Sin850	Singapore, 24 Feb 2004 #
13	AY395003	13-HP03E_ZS-C	Zhongshan, 19 Sep 2003 #	92	AY559082	92-HP03L_Sin852	Singapore, 24 Feb 2004 #
14	AY278488	14-HP03M_BJ01	Beijing, 17 Apr 2003 #	93	AY485277	93-HP03L_Sino1-11	Singapore, 21 Nov 2003 #
15	AY278487	15-HP03M_BJ02	Beijing, 17 Apr 2003 #	94	AY485278	94-HP03L_Sino3-11	Singapore, 21 Nov 2003 #
16	AY278490	16-HP03M_BJ03	Beijing, 17 Apr 2003 #	95	AY559083	95-HP03L_Sin3408	Singapore, 24 Feb 2004 #
17	AY279354	17-HP03M_BJ04	Beijing, 17 Apr 2003 #	96	AY559081	96-HP03L_Sin842	Singapore, 24 Feb 2004 #
18	AY278554	18-HP03M_CUHK-W1	Hong Kong, 17 Apr 2003 #	97	AY559087	97-HP03L_Sin3725V	Singapore, 24 Feb 2004 #
19	AY304495	19-HP03M_GZ50	Guangzhou, 27 May 2003 #	98	AY559088	98-HP03L_SinP1	Singapore, 24 Feb 2004 #
20	AY394977	20-HP03M_GZ-A	Guangzhou, 19 Sep 2003 #	99	AY559089	99-HP03L_SinP2	Singapore, 24 Feb 2004 #
21	AY394993	21-HP03M_HGZ8L2	Guangzhou, 19 Sep 2003 #	100	AY559090	100-HP03L_SinP3	Singapore, 24 Feb 2004 #
22	AY394983	22-HP03M_HSZ2-A	Guangdong, 19 Sep 2003 #	101	AY559091	101-HP03L_SinP4	Singapore, 24 Feb 2004 #
23	AY395004	23-HP03M_HZS2-Bb	Guangdong, 19 Sep 2003 #	102	AY559092	102-HP03L_SinP5	Singapore, 24 Feb 2004 #

24	AY394992	24-HP03M_HZS2-C	Guangdong, 19 Sep 2003 #	103	AY559094	103-HP03L_Sin846	Singapore, 24 Feb 2004 #
25	AY394989	25-HP03M_HZS2-D	Guangdong, 19 Sep 2003 #	104	AY559097	104-HP03L_Sin3408L	Singapore, 24 Feb 2004 #
26	AY394990	26-HP03M_HZS2-E	Guangdong, 19 Sep 2003 #	105	AY568539	105-HP04_GZ0401	Guangzhou, 22 Dec 2003 *
27	AY394987	27-HP03M_HZS2-Fb	Guangdong, 19 Sep 2003 #	106	AY613947	106-HP04_GZ0402	Guangzhou, 05 Jan 2004 *
28	AY394991	28-HP03M_HZS2-Fc	Guangdong, 19 Sep 2003 #	107	AY613950	107-PC04_PC4-227	Guangzhou, 05 Jan 2004 *
29	AY394988	29-HP03M_JMD	Guangdong, 19 Sep 2003 #	108	AY686863	108-PC04_A022	Guangzhou, 15 Jul 2004 #
30	AY508724	30-HP03M_NS1	Beijing, 18 Dec 2003 #	109	AY686864	109-PC04_B039	Guangzhou, 15 Jul 2004 #
31	NC_004718	31-HP03L_Tor2	Toronto, 13 Apr 2003 #	110	AY613949	110-PC04_PC4-136	Guangzhou, 05 Jan 2004 *
32	AY427439	32-HP03L_AS	Italy, 02 Oct 2003 #	111	AY613948	111-PC04_PC4-13	Guangzhou, 10 Jan 2004 *
33	AY291315	33-HP03L_Frank	Germany, 06 May 2 #003	112	AY627046	112-PC04_PC4-145	Guangzhou, 17 May 2004 #
34	AY310120	34-HP03L_FRA	Italy, 29 May 2003 #	113	AY627047	113-PC04_PC4-199	Guangzhou, 17 May 2004 #
35	AY278741	35-HP03L_Urbani	Vietnam, 17 Apr 20 03 #	114	AY572034	114-PC04_civet007	Guangzhou, 11 Mar 2004 #
36	AY278491	36-HP03L_HKU-39849	Hong Kong, 18 Apr 2003 #	115	AY572035	115-PC04_civet010	Guangzhou, 11 Mar 2004 #
37	AY323977	37-HP03L_HSR1	Italy, 22 Jul 2003 #	116	AY572038	116-PC04_civet020	Guangzhou, 11 Mar 2004 #
38	AY278489	38-HP03L_GD01	Guangdong, 17 Ap r 2003 #	117	AY515512	117-PC04_HC-SZ-61	Shenzhen, 31 Dec 2003 #
39	AY313906	39-HP03L_GD69	Guangdong, 03 Jun 2003 #	118	AY545917	118-PC04_HC-GZ-81	Guangzhou, 11 Feb 2004 #
40	AY394978	40-HP03L_GZ-B	Guangdong, 19 Sep 2003 #	119	AY545918	119-PC04_HC-GZ-32	Guangzhou, 11 Feb 2004 #
41	AY394979	41-HP03L_GZ-C	Guangdong, 19 Sep 2003 #	120	AY545914	120-PC04_HC-SZ-79	Shenzhen, 11 Feb 2004 #
42	AY394980	42-HP03L_GZ-D	Guangdong, 19 Sep 2003 #	121	AY545915	121-PC04_HC-SZ-DM1	Shenzhen, 11 Feb 2004 #
43	AY463059	43-HP03L_SH-QXC1	Shanghai, 11 Nov 2003 #	122	AY545916	122-PC04_HC-SZ-266	Shenzhen, 11 Feb 2004 #
44	AY463060	44-HP03L_SH-QXC2	Shanghai, 11 Nov 2003 #	123	AY545919	123-CFB04_SZ	Shenzhen, 11 Feb 2004 #
45	AY461660	45-HP03L_SoD	Russia, 31 Oct 2003 #	124	DQ648856	124-Bat_SARS-273	Hubei, 07 Nov 2004 *
46	AY345986	46-HP03L_CUHK-AG01	Hong Kong, 18 Jul 2003 #	125	DQ648857	125-Bat_SARS-279	Hubei, 08 Nov 2004 *
47	AY345987	47-HP03L_CUHK-AG02	Hong Kong, 18 Jul 2003 #	126	DQ022305	126-Bat_SARS-HKU3	Hong Kong, 29 Apr 2005 #
48	AY345988	48-HP03L_CUHK-AG03	Hong Kong, 18 Jul 2003 #	127	DQ071615	127-Bat_SARS-Rp3	Guangxi, 11 Dec 2004 *
49	AY394998	49-HP03L_CUHK-LC1	Guangdong, 19 Sep 2003 #	128	DQ412042	128-Bat_SARS-Rf1	Hubei, 07 Nov 2004 *
50	AY394999	50-HP03L_CUHK-LC2	Guangdong, 19 Sep 2003 #	129	DQ412043	129-Bat_SARS-Rm1	Hubei, 08 Nov 2004 *
51	AY395000	51-HP03L_CUHK-LC3	Guangdong, 19 Sep 2003 #	Partial genome			

52	AY395001	52-HP03L_CUHK-LC4	Guangdong, 19 Sep 2003 #	130	AY304487	130-PC03_SZ13	Shenzhen, 07 May 2003 *
53	AY395002	53-HP03L_CUHK-LC5	Guangdong, 19 Sep 2003 #	131	AY304489	131-PC03_SZ1	Shenzhen, 07 May 2003 *
54	AY282752	54-HP03L_CUHK-Su10	Hong Kong, 12 Nov 2003 #	132	AY525636	132-HP04_GD03T13	Guangzhou, 13 Jan 2004 #
55	AY350750	55-HP03L_PUMC01	Beijing, 24 Jul 2003 #	133	AY627044	133-PC04_PC4-115	Guangzhou, 17 May 2004 #
56	AY357075	56-HP03L_PUMC02	Beijing, 31 Jul 2003 #	134	AY627045	134-PC04_PC4-137	Guangzhou, 17 May 2004 #
57	AY357076	57-HP03L_PUMC03	Beijing, 31 Jul 2003 #	135	AY613952	135-PC04_PC4-205	Guangzhou, 03 May 2004 #
58	AY338174	58-HP03L_TC1	Taiwan, 28 Jul 2003 #	136	AY627048	136-PC04_PC4-241	Guangzhou, 17 May 2004 #
59	AY338175	59-HP03L_TC2	Taiwan, 28 Jul 2003 #	137	AY613951	137-PC04_PC127	Guangzhou, 03 May 2004 #
60	AY348314	60-HP03L_TC3	Taiwan, 23 Jul 2003 #	138	AY687354	138-PC04_A001	Guangzhou, 16 Jul 2004 #
61	AY394850	61-HP03L_WHU	Wuhan, 12 Jan 2004 #	139	AY687355	139-PC04_A013	Guangzhou, 16 Jul 2004 #
62	AY321118	62-HP03L_TWC	Taiwan, 11 Jun 2003 #	140	AY687356	140-PC04_A021	Guangzhou, 16 Jul 2004 #
63	AY291451	63-HP03L_TW1	Taiwan, 06 May 2003 #	141	AY687373	141-RD04_A022	Guangzhou, 16 Jul 2004 #
64	AY502925	64-HP03L_TW2	Taiwan, 15 Dec 2003 #	142	AY687357	142-PC04_A030	Guangzhou, 16 Jul 2004 #
65	AY502926	65-HP03L_TW3	Taiwan, 15 Dec 2003 #	143	AY687358	143-PC04_A031	Guangzhou, 16 Jul 2004 #
66	AY502927	66-HP03L_TW4	Taiwan, 15 Dec 2003 #	144	AY687359	144-PC04_B012	Guangzhou, 16 Jul 2004 #
67	AY502928	67-HP03L_TW5	Taiwan, 15 Dec 2003 #	145	AY687360	145-PC04_B024	Guangzhou, 16 Jul 2004 #
68	AY502929	68-HP03L_TW6	Taiwan, 15 Dec 2003 #	146	AY687361	146-PC04_B029	Guangzhou, 16 Jul 2004 #
69	AY502930	69-HP03L_TW7	Taiwan, 15 Dec 2003 #	147	AY687362	147-PC04_B033	Guangzhou, 16 Jul 2004 #
70	AY502931	70-HP03L_TW8	Taiwan, 15 Dec 2003 #	148	AY687364	148-PC04_B040	Guangzhou, 16 Jul 2004 #
71	AY502932	71-HP03L_TW9	Taiwan, 15 Dec 2003 #	149	AY687365	149-PC04_C013	Guangzhou, 16 Jul 2004 #
72	AY502923	72-HP03L_TW10	Taiwan, 15 Dec 2003 #	150	AY687366	150-PC04_C014	Guangzhou, 16 Jul 2004 #
73	AY502924	73-HP03L_TW11	Taiwan, 15 Dec 2003 #	151	AY687367	151-PC04_C017	Guangzhou, 16 Jul 2004 #
74	AY362698	74-HP03L_TWC2	Taiwan, 05 Aug 2003 #	152	AY687368	152-PC04_C018	Guangzhou, 16 Jul 2004 #
75	AY362699	75-HP03L_TWC3	Taiwan, 05 Aug 2003 #	153	AY687369	153-PC04_C019	Guangzhou, 16 Jul 2004 #
76	AP006557	76-HP03L_TWH	Taiwan, 30 Jul 2003 #	154	AY687370	154-PC04_C025	Guangzhou, 16 Jul 2004 #
77	AP006558	77-HP03L_TWJ	Taiwan, 30 Jul 2003 #	155	AY687371	155-PC04_C028	Guangzhou, 16 Jul 2004 #
78	AP006559	78-HP03L_TWK	Taiwan, 30 Jul 2003 #	156	AY687372	156-PC04_C029	Guangzhou, 16 Jul 2004 #

* Sampling date for these isolates

Actual date for these sequences submitting to NCBI
