# Supplementary figures and images for: Differential stepwise evolution of SARS coronavirus functional proteins in different host species
Source: BMC Evol Biol. 2009 Mar 5;9:52. doi: 10.1186/1471-2148-9-52 (PMC2676248; doi:10.1186/1471-2148-9-52)

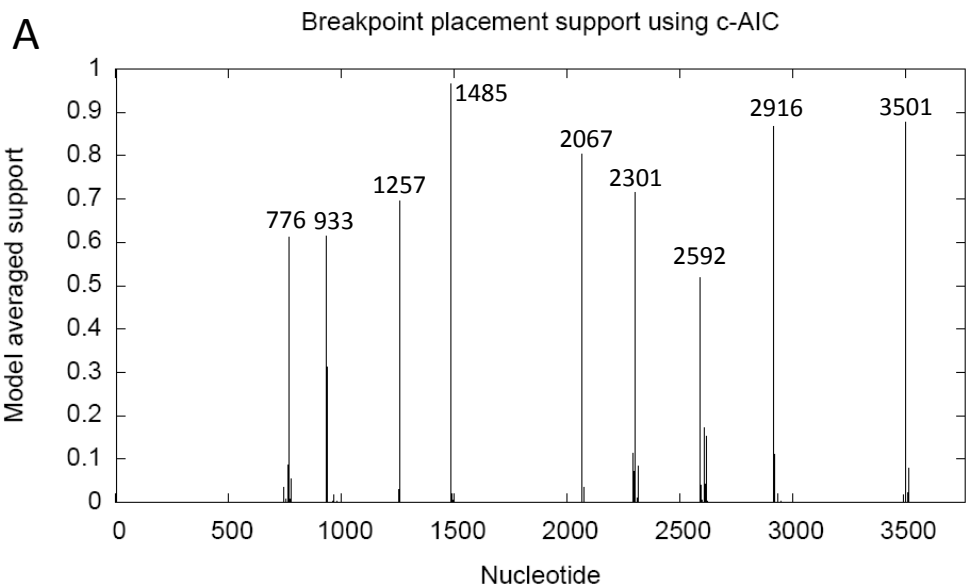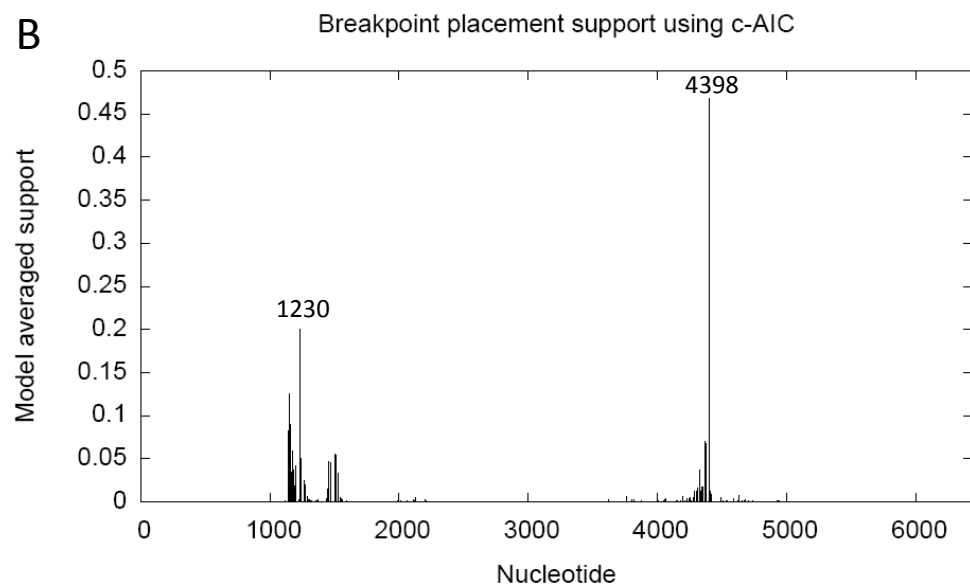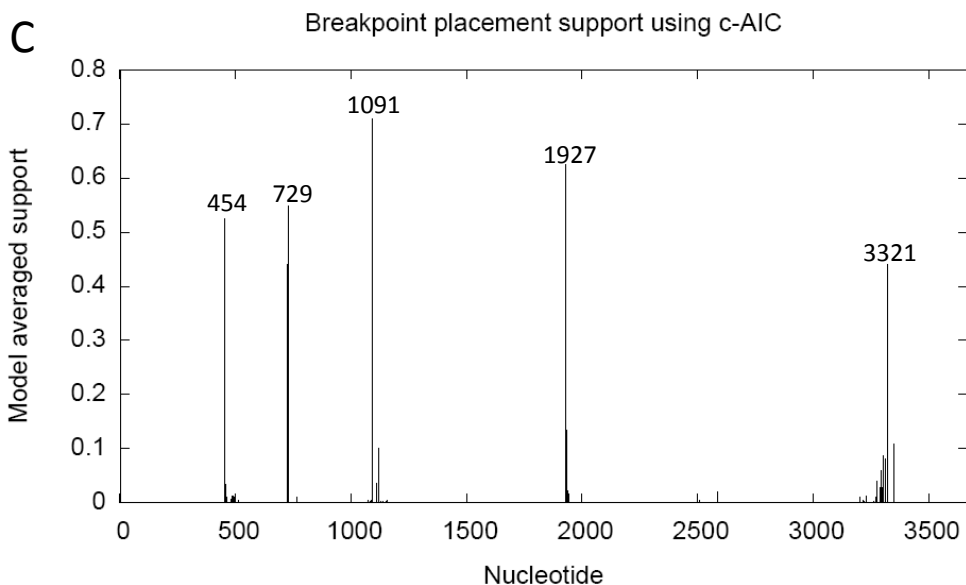

Supplement: Additional file 2 — Figure S1. Detection of recombination with GARD method. (A) putative breakpoints in spike gene; (B) putative breakpoints in replicase domains; (C) putative breakpoints in 3'-end ORFs. [file 1471-2148-9-52-S2.pdf]

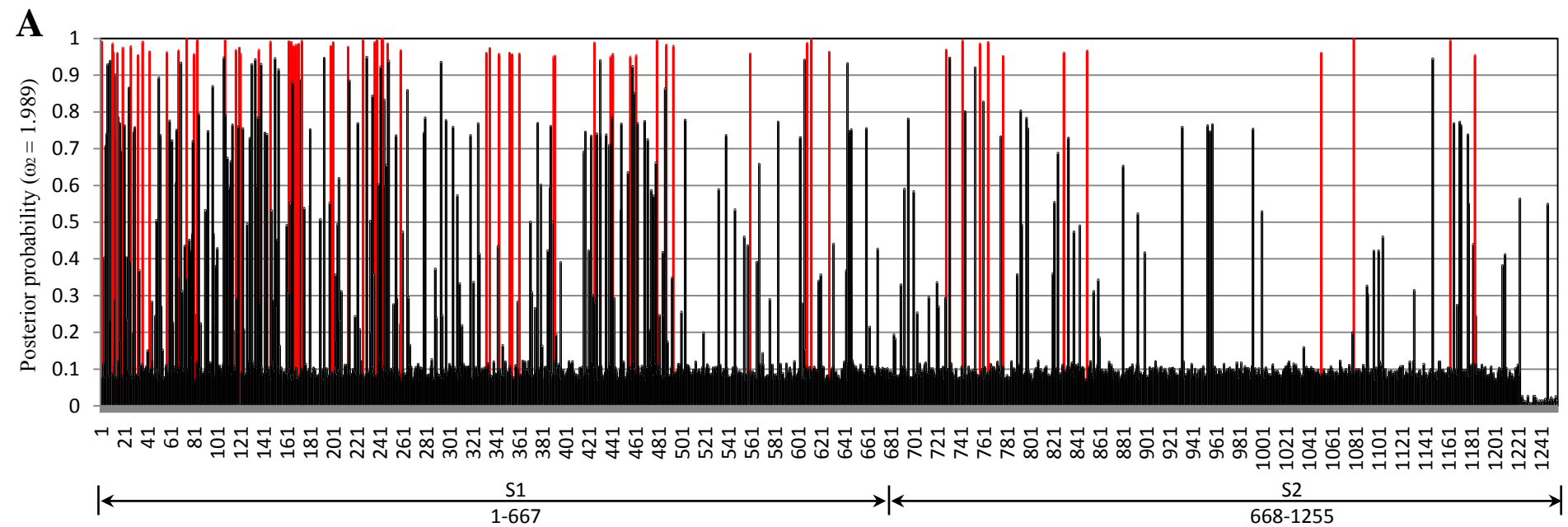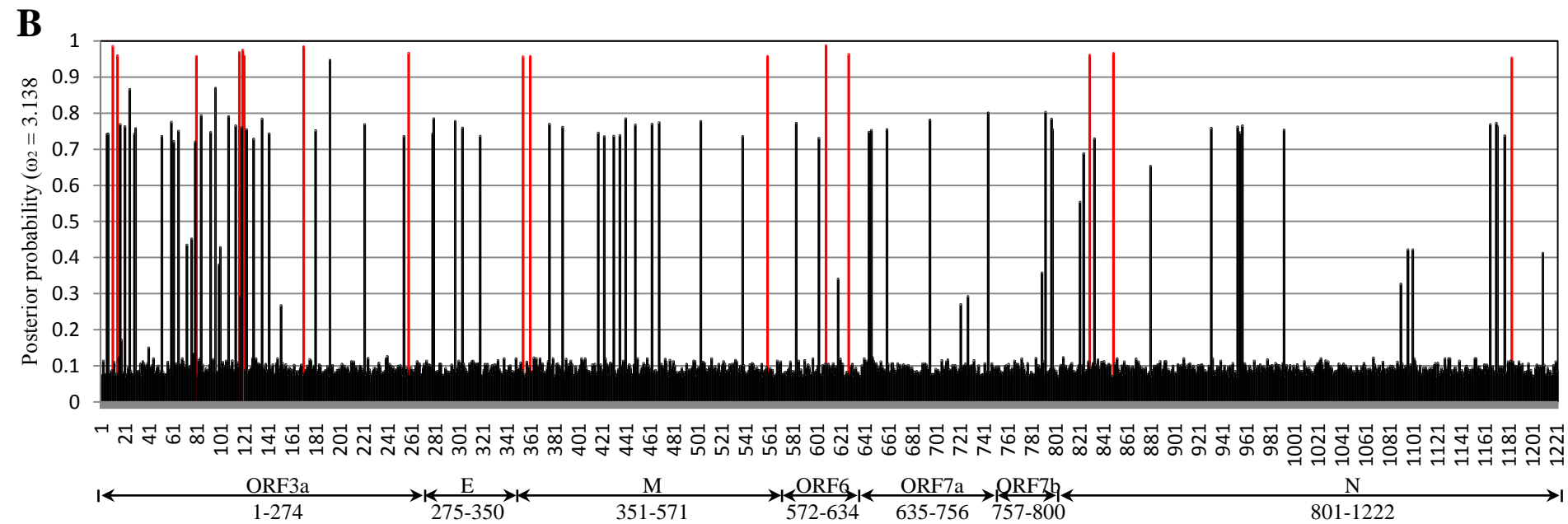

Supplement: Additional file 3 — Figure S2. The distribution of positively selected sites identified using the branch-site model A (SARS group as foreground). (A) Positively selected sites among S protein of SARS-CoV; (B) positively selected sites among 3'-end ORFs of SARS-CoV. The most significant peaks (p >95%) were colored in red. [file 1471-2148-9-52-S3.pdf]
